# A feasibility study evaluating seismocardiography for the detection of heart failure

**DOI:** 10.1371/journal.pdig.0001685

**Published:** 2026-09-10

**Authors:** Ahmad Agam, Emil Korsgaard, Troels Yding Haugstrup, Kasper Janus Grønn Emerek, Maria Weinkouff Pedersen, Massar Omar, Jacob Eifer Møller, Kristian Kragholm, Samuel Emil Schmidt, Peter Søgaard

**Affiliations:** 1 Department of Cardiology, Aalborg University Hospital, Aalborg, Denmark; 2 Department of Clinical Medicine, Aalborg University, AAU, Aalborg, Denmark; 3 Department of Health Science and Technology, Aalborg University AAU, Aalborg, Denmark; 4 Department of Cardiology, Aarhus University Hospital, Aarhus, Denmark; 5 Department of Cardiology, Odense University Hospital, Odense, Denmark; University of Ottawa, CANADA

## Abstract

The study aimed to develop a seismocardiograph (SCG)- based algorithm and assess its diagnostic performance in the detection of heart failure (HF). A total of 218 subjects were included: 198 with suspected HF and 20 with known HF with reduced ejection fraction (HFrEF) were included for testing only. Assessments were conducted using SCG, N-terminal pro b-type natriuretic peptide (NT-proBNP), electrocardiogram, NYHA classification and echocardiography. SCG-based algorithms, “AnyHF score” were developed to identify all subtypes of HF and “HFrEF-score” to identify HFrEF. Diagnostic accuracy was assessed using sensitivity, specificity, positive predictive value (PPV), negative predictive value (NPV), and the area under the receiver-operating characteristic curve (AUC-ROC). The AnyHF score demonstrated an AUC of 82%, sensitivity of 90.9%, specificity of 43.8%, NPV of 87.5% and PPV of 52.6% in detecting HF versus no HF. A balanced comparative analysis was performed between NT-proBNP and the HFrEF-score for detecting HFrEF versus no HF. NT-proBNP demonstrated an AUC of 94.7%, sensitivity 94.1%, specificity 68.8%, NPV 99%, and PPV 27.1%. The HFrEF-score showed an AUC of 92.9%, sensitivity 88.2%, specificity 92% (p < 0.001), NPV 98.4%, and PPV 57.7% (p = 0.007). The SCG-scores also categorized 71 patients (51%) from the no HF group, referred on suspicion of HF, as minimal risk of HF. This study suggests that SCG has potential to aid in the diagnostic process of HF. The SCG-based algorithms “AnyHF-score” and “HFrEF-score” demonstrated high diagnostic performance in identifying HF.

## Author summary

In this study, we explored a new way to detect heart failure using a small device that can measure the vibrations of the chest caused by the beating heart. Heart failure is a common condition in which the heart cannot pump blood effectively, and it can be difficult to diagnose early with standard tests. We developed a computer-based method that analyzes these chest vibrations to identify signs of heart failure and to distinguish between different types of condition.

Our approach may provide a fast, non-invasive, and easy-to-use tool that could support doctors in diagnosing heart failure earlier and more accurately, potentially improving patient care and reducing the need for more invasive or expensive tests.

By showing that chest vibration signals contain important information about heart function, this work opens the door to using simple wearable or bedside devices to screen for heart failure in clinical settings or even in the community. In the future, this could help detect heart problems before they become severe, benefiting patients and healthcare systems alike.

## Introduction

Heart failure (HF) is associated with high morbidity and mortality, and patients are frequently hospitalized with subsequent high health care costs [1–3]. Transthoracic echocardiography (TTE) and brain natriuretic peptide (NT-proBNP) are guideline recommended assessments of patients with suspected HF [4–6]. However, access to diagnostic tools such as TTE and NT-proBNP is limited in many low- and middle-income countries [7,8]. At the same time, healthcare systems in high-income countries are increasingly strained by ageing populations and the growing demand for advanced treatments [9]. TTE has certain issues such as operator-dependent interpretation and long waiting lists that can delay the diagnosis [10–14]. Similarly, there are limitations in the use of NT-proBNP including delay in central lab analysis and the fact that various non-cardiac factors can influence NT-proBNP levels [5]. Both situations show the need for accessible and cost-effective diagnostic solutions that are easy to use and suitable for use outside traditional hospital settings, including primary care facilities and community health centers.

Seismocardiography (SCG) is non-invasive and uses an accelerometer for recording heart-generated vibrations from mechanical cardiac processes such as muscle contraction, valve movement, blood flow turbulence and momentum changes [15]. Through simultaneous SCG and electrocardiogram (ECG) recordings key fiducial points in the SCG waveform have been identified and related to the cardiac events, aortic opening, aortic closing, mitral valve opening and mitral valve closing [16,17]. Studies have demonstrated that SCG measurements correlate with preload changes [18,19] and early diastolic velocity (e’) from tissue-doppler echocardiography [20]. SCG has also proven capable of distinguishing between compensated and decompensated patients with HF [21]. In this study, we evaluated the potential of SCG for HF detection in patients with suspected HF.

The primary aim was to develop and validate SCG-based algorithms for HF detection. Secondary aims were to assess their accuracy for detecting HF with preserved ejection fraction (HFpEF) and to compare algorithm performance with NT-proBNP for identifying HF with reduced ejection fraction (HFrEF).

## Methods

In the current study, patients referred by their general practitioner for suspected HF to Aalborg University Hospital or Odense University Hospital, Denmark, were enrolled. The study is registered at ClinicalTrials.gov (NCT03656354).

### Study population

**Fig 1:**
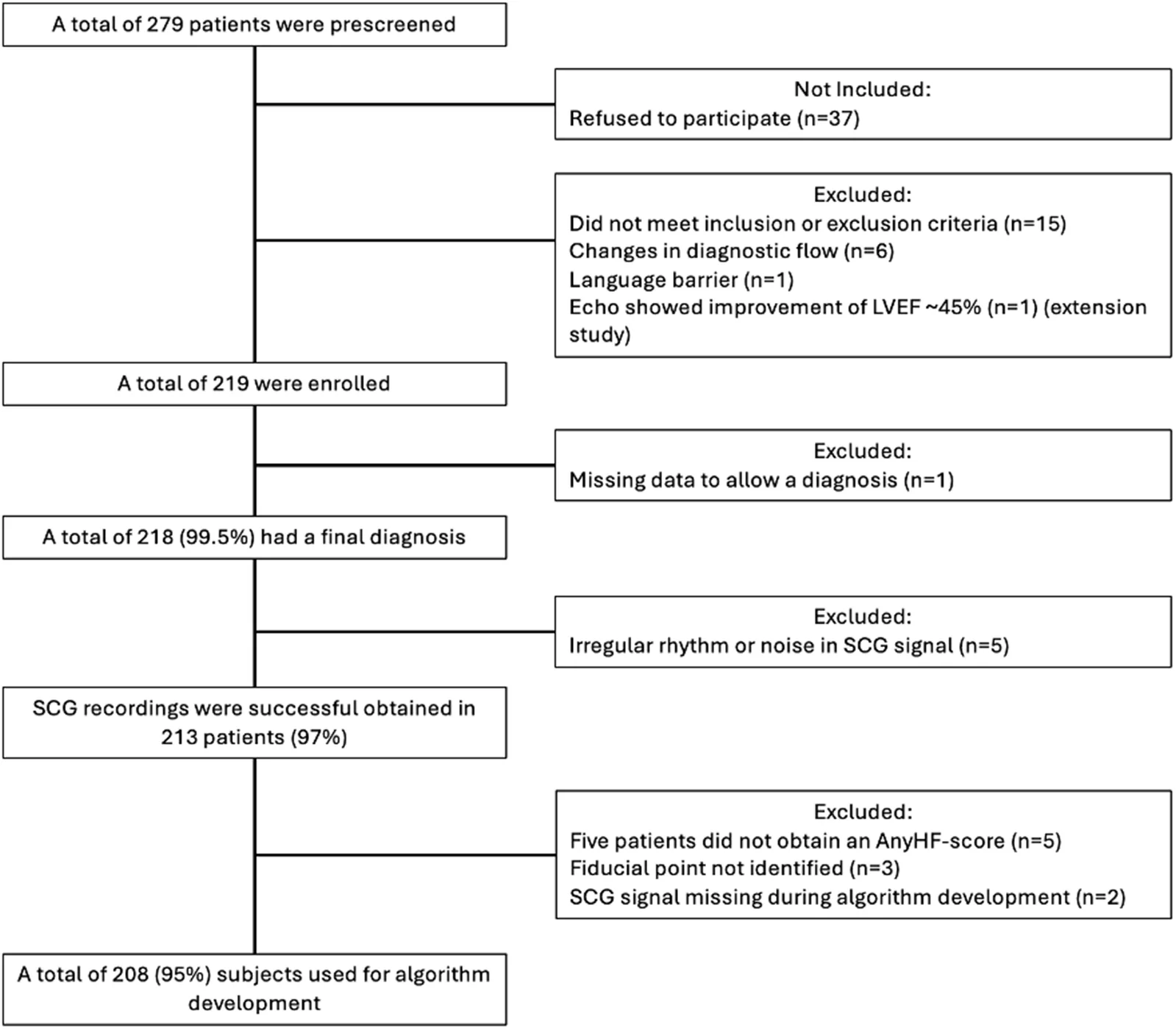
Enrollment flow diagram.

The study population was divided using a predefined sequential 50/50 split based on enrollment order. This allocation was performed prior to model development and was independent of HF subtype or other clinical characteristics. In the study, a total of 218 patients were included with a final HF diagnosis after exclusion criteria. The main study included 198 patients with suspected HF. The main study included only 9 subjects with HFrEF. Therefore, a validation study at Aalborg University Hospital enrolled 20 newly diagnosed subjects with HFrEF to address their underrepresentation in the main study. The validation cohort was collected after completion of algorithm development and used exclusively to validate the performance of the locked algorithms in patients with HFrEF. These patients were therefore not included in model training or algorithm development. Fig 1 shows the enrollment flow diagram.

The inclusion criteria were as follows: males or females aged 18 years or older. They must be able to comply with the clinical investigation plan and provide signed informed consent. The exclusion criteria were: 1) a known diagnosis of HF, 2) atrial fibrillation, or 3) an ongoing or newly diagnosed acute coronary syndrome (ACS) considered to be the primary cause of the current HF episode. Additional exclusion criteria included the presence of an implanted donor heart, permanent left ventricular assist device, as well as the presence of a pacemaker or implantable cardioverter defibrillator. Participants with any other implanted electronic equipment in the area above and around the heart, significant surgical scars in the SCG recording area were excluded. Furthermore, pregnancy was an exclusion criterion.

### Experimental design

All patients underwent a standardized procedure including clinical history, physical examination, ECG, NT-proBNP, and TTE for the assessment of structural heart disease. The SCG recording was performed by the same investigator who conducted the clinical examination, but before TTE and blood sampling, and without knowledge of the results from these subsequent investigations. HF diagnosis was determined according to a predefined standardized procedure (see patient classification section below) and was not influenced by the blinded SCG measurement (S1 Fig in the supplementary materials).

### SCG, ECG and NT-proBNP measurements

SCG recordings were performed with the CADScor SCG device, (Acarix SE), which included an ADXL 354 accelerometer (Analog Devices Inc.,US), sample rate 8000 sps, at two positions, the IC4 and the xiphoid process. The data obtained at the xiphoid process was used for algorithm development and validation. The SCG measurement took place in a quiet room before the resting ECG, blood sampling and TTE were performed. A standardized 12-lead ECG was recorded, and a routine blood sample was collected to measure plasma NT-proBNP levels. In the extension study results from a 12-lead ECG and NT-proBNP measurements were entered if obtained as part of the diagnostic pathway before inclusion in the study.

### Echocardiography

TTE was performed after completion of the SCG analysis and resting ECG by an experienced operator on a GE Healthcare Vivid e95 ultrasound machine (GE Healthcare, Horten Norway). Images were analyzed offline on EchoPAC Version 201 (GE Medical system, Horten, Norway) blinded for SCG data. Standard transthoracic 2D and Doppler images were acquired according to international recommendations [22]. In addition to standard TTE measurements using 2D and Doppler imaging to assess left ventricular (LV) function, LV longitudinal deformation was assessed from speckle tracking analyses using an 18-segment LV model reporting global longitudinal strain (GLS) in absolute value. Left ventricular ejection fraction (LVEF) was measured using AutoEF, and left atrial volume index (LAVI) was assessed by measuring left atrial volume in both the apical 2-chamber and 4-chamber views in end-systole.

### Patient classification

The subjects in this study were divided into HFrEF, HFpEF, HF with mildly reduced ejection fraction (HFmEF) and no HF and the following classification was used:

Patients were classified as having HFrEF if both of the following criteria were met:

1. Presence of HF symptoms and/or signs, including dyspnoea, orthopnoea, paroxysmal nocturnal dyspnoea, reduced exercise tolerance, fatigue, and ankle swelling
2. LVEF <40%.

Patients were classified as HFmEF or HFpEF if all four of the following criteria were met:

1. Presence of HF symptoms and/or signs, including dyspnoea, orthopnoea, paroxysmal nocturnal dyspnoea, reduced exercise tolerance, fatigue, and ankle swelling
2. Preserved LVEF (≥40%).
3. Elevated NT-proBNP levels (>125 pg/mL).
4. Objective evidence of structural or functional cardiac abnormalities, including:
  - LAVI >34 mL/m²
  - LV mass index ≥115 g/m² (males) or ≥95 g/m² (females)
  - E/e′ ≥ 13 (average e’) and mean e′ (septal and lateral) <9 cm/s

Patients with LVEF ≥50% were classified as having HFpEF and those with LVEF in the range of 40–49% were classified as HFmEF.

### Algorithm development

Two algorithms were developed: the “AnyHF-score” to detect the probability of having either HFrEF, HFmEF, or HFpEF and the “HFrEF-score” to specifically estimate the probability of HFrEF. The AnyHF-score was trained and evaluated on subjects with and without HF. The training set consisted of the first 100 subjects (75 without HF and 25 with HF), while the test set included the rest 108 subjects (64 without HF and 44 with HF). The HFrEF-score was trained in 79 subjects (75 without HF and 4 with HFrEF), and it was evaluated on 89 subjects (64 with no HF and 25 with HFrEF). The 20 patients with known HFrEF from the extension study were used in the test set only. The split of the training and validation sets was performed according to the study protocol.

Since HF can be caused by many different underlying conditions, it is expected that HF can alter the SCG signal in many ways. Thus, simple amplitude and time interval measures might not capture HF, and it is not expected that a distinct HF morphology can be identified in the SCG signal. Therefore, the current method aims at detecting abnormalities in the SCG signal as an indicator of HF. Autoencoders were utilized to detect abnormalities in the systolic and diastolic SCG signals. Auto-encoders are artificial neural networks that can be applied to encode signals known to the network down to a very compact form, from which the signal can be reconstructed.

Using an existing algorithm developed for the segmentation of heart sounds [23] the SCG was segmented into systolic and diastolic segments, from which an ensemble average systolic interval and an ensemble average diastolic interval were estimated. For both intervals, an autoencoder was trained in SCG intervals from patients without any HF. Thereby, the autoencoders are trained to reconstruct SCG waves from patients without HF but not trained to reconstruct SCG waves from HF patients. The autoencoder’s reconstruction performance was quantified using the Pearson correlation coefficient between the input SCG signal and its reconstructed output, see Graphical Abstract**.** A detailed description of the algorithm development is provided in S1 Text in the supplementary material. The HFrEF-score was estimated using a logistic regression, including only the two reconstruction measures from the systolic (rSys) and diastolic (rDia) signals. While the Any HF-score was estimated as a logistic regression including the two reconstructi measures, age, sex, the diastolic amplitudes as defined by Sørensen et al. [17]

### Statistical analysis

#### Analysis of primary endpoint.

The primary objective was to develop an SCG-based algorithm for detecting HF and assess its diagnostic performance. The primary endpoints included the area under the receiver-operating characteristic curve (AUC-ROC), sensitivity, specificity, and positive and negative predictive values (PPV, NPV) for detecting HF. SCG recordings from patients with HF and without HF were analyzed to identify distinguishing parameters, such as the amplitude and time intervals. The parameters were combined into a continuous score using an appropriate classification method like a linear discriminant function, a logistic regression, or a support vector machine. The AUC-ROC was calculated using SCG-readouts as continuous variables, while sensitivity, specificity, PPV, and NPV were calculated after dichotomizing the score at a cutoff of 0.15. All performance metrics were reported with 95% confidence intervals. The resulting algorithm, termed “AnyHF-score”, provided a continuous output for HF detection.

### Analysis of secondary endpoints

A secondary objective was to assess the diagnostic accuracy of SCG and NT-proBNP in detecting HFrEF. The endpoints included AUC-ROC, sensitivity, specificity, PPV and NPV. These were calculated using continuous variables for the AUC-ROC calculations, and dichotomized SCG-readouts. An algorithm, termed the “HFrEF-score,” was developed to provide a continuous score for HFrEF detection, dichotomized at a cutoff of 0.05. NT-proBNP values were dichotomized using a threshold of 125 pg-mL. These calculations were performed for both patients with HFrEF and no HF. The final secondary objective was to assess the diagnostic accuracy of the SCG-based AnyHF-score in detecting HFpEF. AUC-ROC, sensitivity, specificity, PPV, and NPV were calculated for SCG using dichotomized readouts, similarly to the primary endpoint analysis, comparing HFpEF patients with no HF.

The probabilistic thresholds (AnyHF: 0.015; HFrEF: 0.05) were predefined during algorithm development based on the intended clinical use and an accepted level of false negative risk, rather than being optimized against ROC-derived performance metrics in the independent test cohort.

#### Definition of data sets included in the statistical analyses.

**Performance analysis set for SCG:** all subjects not included in the algorithm development and having both a SCG-readout and an outcome from the HF examinations (patients with HFrEF, HFmEF, HFpEF, or no HF).

**Performance analysis set for NT-proBNP:** all subjects have both an NT-proBNP value and the diagnosis HFrEF or being categorized as no HF.

### Ethics

The study was approved by the North Denmark Region Committee on Health Research Ethics (N-20170090) and conducted in accordance with the Declaration of Helsinki. The subjects signed a written informed consent before they participated in the study. All methods were performed in accordance with relevant guidelines and regulations.

## Results

### Baseline characteristics

The study population consisted of 218 patients (Table 1). SCG recordings from 11 patients were excluded due to data quality issues, leaving 208 subjects for algorithm development and validation. The baseline demographic characteristics of the population are shown in Table 1. The population was characterized by a mean age of 66 years, 51% woman and a mean body mass index of 29.8. A total of 133 (61%) subjects had a history of hypertension, and 25 (11%) subjects had a history of coronary artery disease (CAD). The supplementary material presents the BNP and TTE measurement data for the study participants in S2 **Table** and S3 **Table**.

**Table 1:** Baseline characteristics.

	All	No HF	HFrEF	HFmEF	HFpEF
**Age (years), N**	**218**	**145**	**30**	**4**	**39**
**Mean ± SD**	66.3 ± 11.7	65.4 ± 11.1	60.8 ± 13.6	79.5 ± 5.8	72.2 ± 8.7
**Min-Max**	20 – 89	20 – 88	30 – 89	74 – 85	51 – 87
**Sex, N (%)**	**218**	**145**	**30**	**4**	**39**
**Female**	111 (50.7)	67 (48.9)	12 (40.0)	2 (50.0)	21 (58.3)
**Ethnicity, N (%)**	**218**	**145**	**30**	**4**	**39**
**White**	215 (98.2)	137 (100.0)	28 (93.3)	4 (100.0)	35 (97.2)
**Other**	4 (1.8)	0 (0.0)	2 (6.7)	0 (0.0)	1 (2.8)
**Weight (kg), N**	**208**	**136**	**30**	**4**	**37**
**Mean ± SD**	87.4 ± 18.5	87.1 ± 16.9	83.6 ± 21.2	85.8 ± 21.8	91.9 ± 21.6
**Min-Max**	47 – 150	47 – 134	52 – 146	71 – 118	60 – 150
**BMI, N**	**208**	**136**	**30**	**4**	**37**
**Mean ± SD**	29.8 ± 5.6	29.9 ± 5.3	27.6 ± 5.2	29.1 ± 8	31.3 ± 6.5
**Min-Max**	17.9 – 51	17.9 – 50.8	21.1 – 47.1	22.4 – 39	20.1 – 51
**Abdominal circumference, N**	**211**	**139**	**29**	**4**	**38**
**Mean ± SD**	105.9 ± 16	106.2 ± 14.5	98 ± 14	108.8 ± 17.6	110.3 ± 20.3
**Min-Max**	52 – 166	71 – 145	80 – 140	93 – 133	52 – 166
**History of CAD, N (%)**	**218**	**145**	**30**	**4**	**39**
**Yes**	25 (11.4)	18 (12.4)	1 (3.3)	2 (50.0)	4 (10.3)
**History of hypertension, N (%)**	**218**	**145**	**30**	**4**	**39**
**Yes**	133 (60.7)	90 (62.1)	11 (36.7)	4 (100.0)	28 (71.8)
**Smoking, N (%)**	**218**	**145**	**30**	**4**	**39**
**Current smoker**	29 (13.2)	15 (10.3)	8 (26.7)	0 (0.0)	6 (15.4)
**Former smoker**	113 (51.6)	76 (52.4)	8 (26.7)	4 (100.0)	25 (64.1)
**Never smoker**	77 (35.2)	54 (37.2)	14 (46.7)	0 (0.0)	8 (20.5)
**Diabetes mellitus, N (%)**	**218**	**145**	**30**	**4**	**39**
**No**	173 (79.0)	120 (82.8)	21 (70.0)	3 (75.0)	28 (71.8)
**Type I**	5 (2.3)	3 (2.1)	1 (3.3)	0 (0.0)	1 (2.6)
**Type II**	41 (18.7)	22 (15.2)	8 (26.7)	1 (25.0)	10 (25.6)
**NYHA, N (%)**	**72**		**30**	**4**	**39**
I	9 (11)		1 (3.3)	0 (0.0)	7 (17.9)
II	35 (47.9)		12 (40.0)	2 (50.0)	21 (53.8)
III	25 (34.2)		13 (43.3)	2 (50.0)	10 (25.6)
IIII	5 (6.8)		4 (13.3)	0 (0.0)	1 (2.6)

### Development of seismocardiographic scores

**S1 Table** from the supplementary presents the AnyHF-score and HFrEF score in all patient groups. AnyHF-score outputs a continuous value, dichotomized by a cut-off of 0.15. The mean AnyHF-scores (±SD) for patients in distinct groups: No HF: 0.219 ± 0.221, HFpEF: 0.497 ± 0.283 and HFrEF: 0.733 ± 0.277. The HFrEF-score also produces a continuous value; however, it is dichotomized by a cut-off of 0.05. The mean HFrEF scores (±SD) for patients in distinct groups: No HF: 0.0212 ± 0.0616, HFpEF: 0.0558 ± 0.101 and HFrEF: 0.384 ± 0.292

### Performance of AnyHF-score in detecting all HF and HFpEF

**Fig 2:**
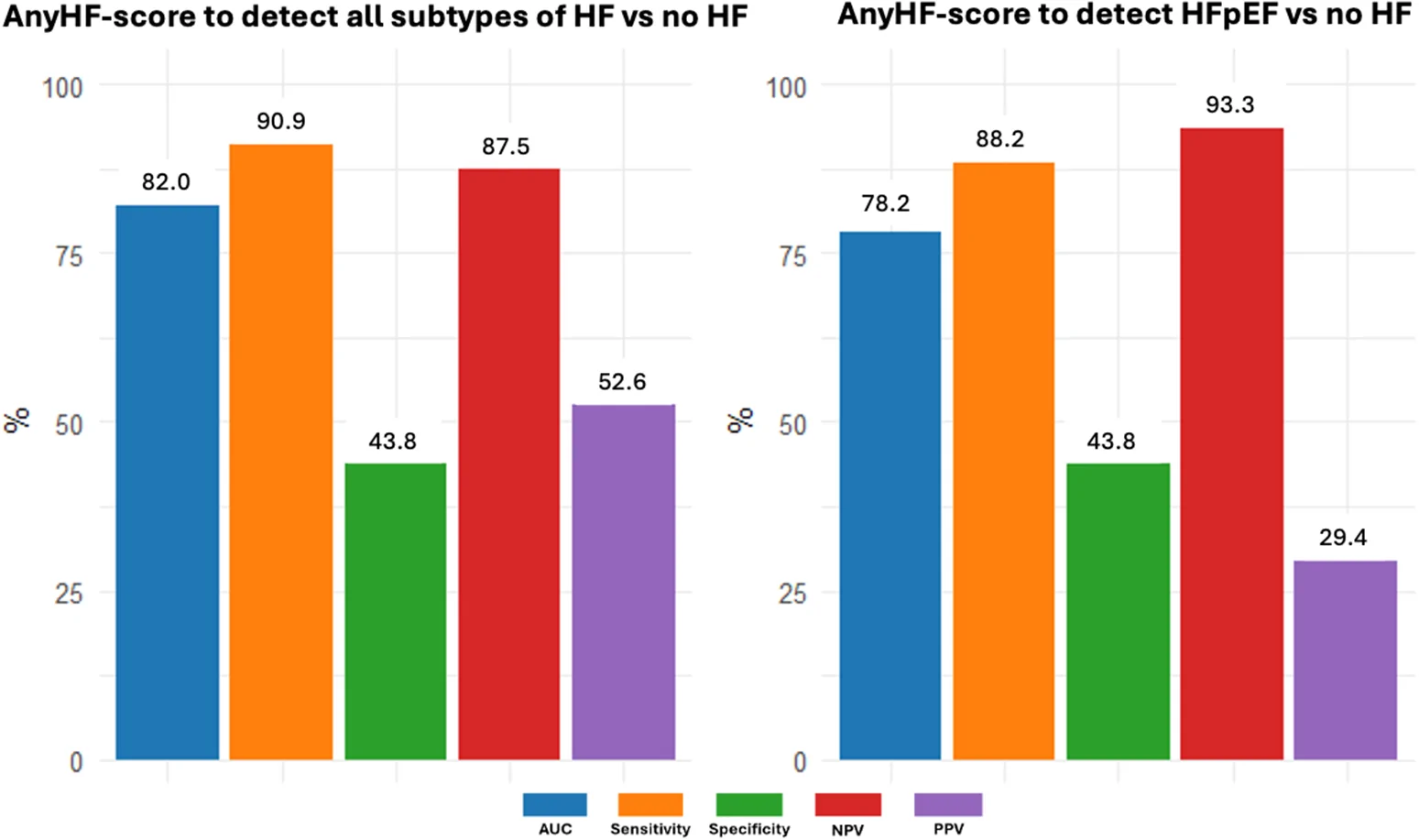
Presents the performance of the SCG-based AnyHF-score in detecting all types of HF and HFpEF specifically in the test group. HF is defined as HFrEF, HFmEF and HFpEF combined. Negative predictive values, specificity are calculated for ‘AnyHF-scores’ ≤0.15. Positive predictive values and sensitivity are calculated for ‘AnyHF-scores’ >0.15. CI: 95% confidence interval.

Fig 2 presents the performance of the AnyHF-score to detect all types of HF versus no HF and HFpEF vs no HF. The AnyHF-score showed an AUC of 82%, with a sensitivity of 90.9%, specificity of 43.8%, NPV of 87.5%, and PPV of 52.6% in detecting all types of HF. The Any HF-score showed an AUC of 78.2%, with a sensitivity of 88.2%, specificity of 43.8%, NPV of 93.3%, and PPV of 29.4% in detecting HFpEF vs no HF. A more detailed presentation of these metrics, including additional statistical measures, can be found in S4 **Table** and S5 **Table** in the supplementary. S2 **Fig** in the supplementary presents the ROC-AUC curve of AnyHF-score to detect all HF vs no HF and HFpEF vs no HF.

### Performance of HFrEF-score and NT-proBNP in detecting all HFrEF

**Fig 3:**
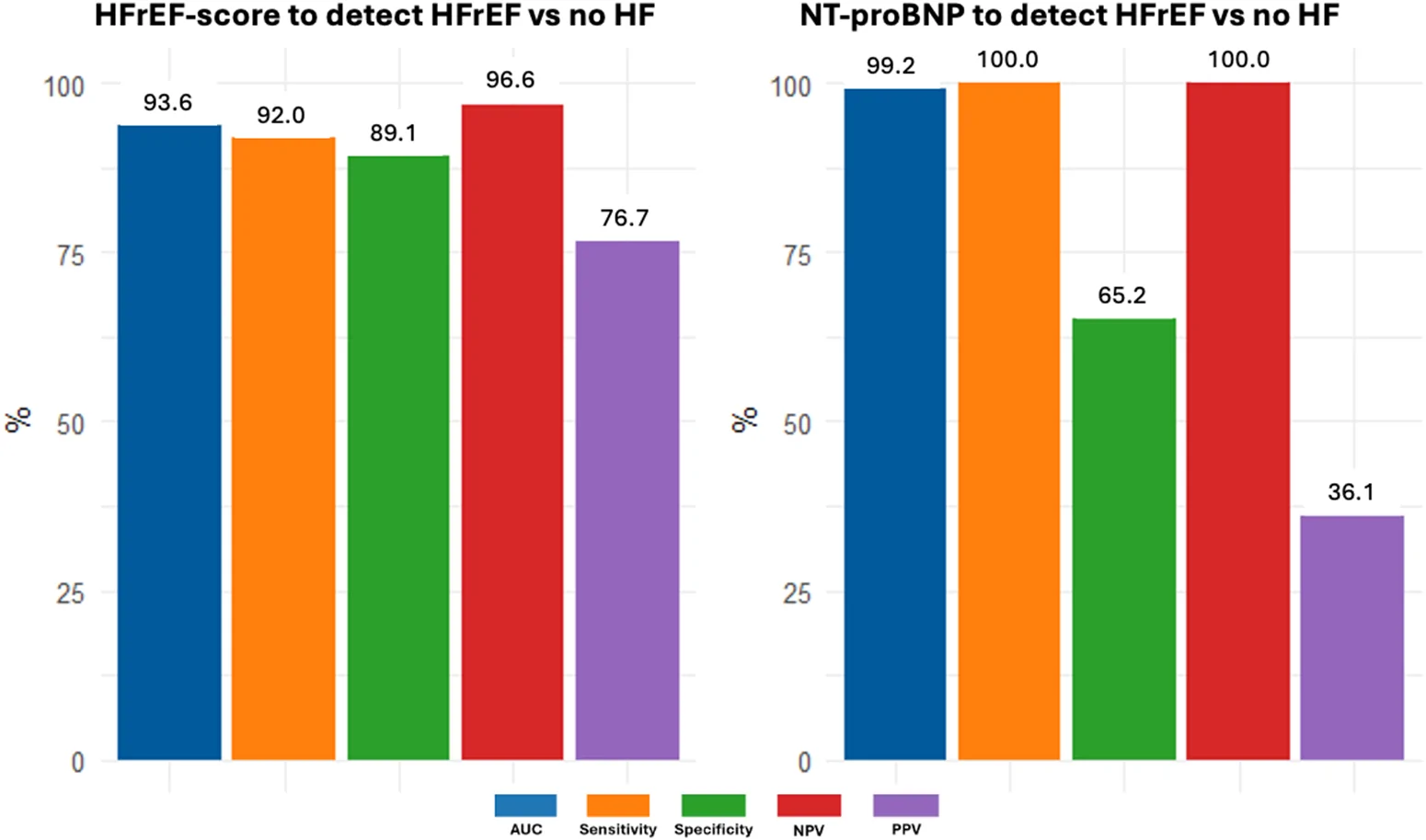
Presents the performance of the SCG-based HFrEF-score and NT-proBNP in detecting HFrEF vs no HF specifically in the test group. Negative predictive values, specificity, are calculated for ‘HFrEF-score’ ≤0.05. Positive predictive values, sensitivity, rule-in and likelihood ratio positive are calculated for ‘HFrEF-score’ >0.05. CI: 95% confidence interval. Negative predictive values, specificity, are calculated for pro-BNP ≤ 125pg-ml. Positive predictive values and sensitivity are calculated for pro-BNP > 125pg-ml. CI: 95% confidence interval.

Fig 3 presents the performance of the HFrEF-score and NT-proBNP in detecting HFrEF versus no HF. The HFrEF-score showed an AUC of 93.6%, with a sensitivity of 92%, specificity of 89.1%, NPV of 96.6%, and PPV of 76.7% in detecting HFrEF vs no HF. NT-proBNP demonstrated an AUC of 99.2%, with a sensitivity of 100%, specificity of 65.2%, NPV of 100%, and PPV of 36.1%. A more detailed presentation of these metrics, including additional statistical measures, can be found in S6 **Table** and **S7 Table** in the Supplementary. S3 Fig in the Supplementary presents the ROC-AUC curves of HFrEF-score and NT-proBNP for detecting HFrEF vs no HF. These results represent the full, unbalanced cohort. To further assess the robustness of the developed algorithms, an additional 5-fold cross-validation was performed on the development cohort and demonstrated similar discrimination across all classification tasks (Supplementary S8 Table). Site-specific analyses further showed comparable performance of the HFrEF-score and AnyHF-score across the Aalborg and Odense study sites (Supplementary S10 and S11 Tables). Balanced Comparative Analysis of NT-proBNP and HFrEF-score

**Table 2** presents a balanced comparative analysis of the performance of NT-proBNP and HFrEF-score in detecting HFrEF versus no HF. The balanced dataset was created to make the comparison more representative and comparable by ensuring that the populations were of similar size and distribution. This analysis provides a more direct comparison between the two methods. All subjects not included in the SCG algorithm development and having both a SCG readout and NT-proBNP value as well as a diagnosis of HFrEF or no HF were included. NT-proBNP demonstrated an AUC of 94.7%, with a sensitivity of 94.1%, specificity of 68.8%, NPV of 99%, and PPV of 27.1%. In contrast, the HFrEF-score showed an AUC of 92.9% (p = 0.742), sensitivity of 88.2% (p = 0.564), specificity of 92% (p < 0.001), NPV of 98.4%, and PPV of 57.7% (p = 0.007).

**Table 2:** Balanced analysis: performance of NT-proBNP and HFrEF-score to detect HFrEF versus No HF.

	NT-proBNP	HFrEF-score	p-values
**No HF, N**	138	138	
**HfrEF, N**	17	17	
**Prevalence of HFrEF, %**	11%	11%	
**True negative**	95	127	
**False negative**	1	2	
**False positive**	43	11	
**True positive**	16	15	
**AUC, % (CI)**	94.7% (87.2-100%)	92.9% (84.3-100%)	0.742
**Sensitivity, % (CI)**	94.1% (71.3-99.9%)	88.2% (63.6-98.5%)	0.564
**Specificity, % (CI)**	68.8% (60.4-76.4%) *	92% (86.2-96%) *	<0.001
**Negative predictive value, % (CI)**	99% (94.3-100%)	98.4% (94.5-99.8%)	0.742
**Positive predictive value, % (CI)**	27.1% (16.4-40.3%) *	57.7% (36.9-76.6%) *	0.007
**Rule out, % (CI)**	61.9% (53.8-69.6%) *	83.2% (76.4-88.7%) *	<0.001
**Rule in, % (CI)**	38.1% (30.4-46.2%) *	16.8% (11.3-23.6%) *	<0.001
**Likelihood ratio positive**	3.021	11.07	
**Likelihood ratio negative**	0.08545	0.1278	

Table 2: Negative predictive values, specificity, rule-out and likelihood ratio negative are calculated for the SCG-based ‘HFrEF-score’ ≤ 0.05. Positive predictive values, sensitivity, rule-in and likelihood ratio positive are calculated for ‘HFrEF-score’ > 0.05. Negative predictive values, specificity, rule-out and likelihood ratio negative are calculated for pro-BNP ≤ 125pg-ml. Positive predictive values, sensitivity, rule-in and likelihood ratio positive are calculated for pro-BNP > 125pg-ml. CI: 95% confidence interval *Comparisons with p-value < 0.05: Specificity, PPV and rule-in-rule-out.

### Risk categories based on AnyHF and HFrEF scores

Risk categories derived from the two SCG-algorithms are illustrated in S4 **Fig**, with three risk groups defined using specific cut-offs:” “Minimal risk of HF”,” Elevated risk of HF”, and ”Elevated risk of low LVEF”. The total sample included 208 subjects and is presented in Table 3.

**Table 3:** Risk categories based on the two SCG scores.

	No HF	HFrEF	HFpEF
**Total, N (%)**	139 (100%)	29 (100%)	36 (100%)
**Minimal risk of HF**	71 (51%)	1 (3%)	5 (14%)
**Elevated risk of HF**	57 (41%)	2 (7%)	21 (58%)
**Elevated risk of low EF**	11 (8%)	26 (90%)	10 (28%)

## Discussion

In this exploratory study, two SCG-based models for HF detection were developed: one for identifying any HF type and another specifically for HFrEF. Although the dataset was limited, both models demonstrated high and promising diagnostic potential. The AnyHF score achieved high sensitivity but modest specificity, while the HFrEF-score showed more high performance comparable to NT-proBNP with good discriminative ability for detecting HFrEF.

### Seismocardiography detection of Heart Failure

This study demonstrates that SCG-based assessment of cardiac function has the potential to identify HF. These findings are consistent with the results from *Agam et al* [19,20], who demonstrated that the SCG could identify diastolic dysfunction and preload changes and potentially patients with HF. The study by *Haddad et al.* [24] demonstrated that smartphone-based assessment of cardiac function using SCG is feasible in identifying and differentiating patients with HF from control subjects with high diagnostic accuracy. The findings from *Haddad et al*. [24] indicate that SCG has the potential to identify HF, thus aligning with the results from this study, however in this study SCG accelerometers were used instead of a smartphone. In this study, the HFrEF-score demonstrated better performance than the AnyHF-score, which was used to detect all subtypes of HF. This is likely because HFrEF is easier to distinguish from a normal SCG, as the reduced cardiac function in patients with HFrEF results in more pronounced abnormalities in the SCG signal. However, given the relatively small training and test cohorts and the absence of external validation, the reported diagnostic performance should be considered exploratory.

### Diagnosis of HFpEF

Diagnosing HFpEF is challenging both clinically and with TTE [25]. In this study, the AnyHF-score showed high AUC, sensitivity, and NPV for detecting HFpEF versus no HF, indicating its potential as a supportive diagnostic tool. SCG may provide additional hemodynamic and mechanical insights that complement existing methods. However, its low PPV and the small HFpEF subgroup highlight that these findings are highly exploratory and should be interpreted with caution.

### Comparison of SCG and NT-proBNP for HF diagnosis

NT-proBNP demonstrated high AUC, sensitivity, and NPV for identifying HFrEF, making it reliable for ruling out HF. However, its PPV is notably low, making it poor at ruling in HF. This is a known problem with NT-proBNP and has been shown by several studies [4,5]. Compared with NT-proBNP, the SCG-based HFrEF-score showed similar AUC, sensitivity, and NPV, but significantly higher specificity and PPV, suggesting that SCG has the potential to be more effective for confirming HFrEF. Although the sensitivity of the HFrEF-score was slightly lower than that of NT-proBNP, this difference was not statistically significant. From a clinical perspective, the higher specificity and PPV of SCG compared to NT-proBNP could complement NT-proBNP and reduce false-positive cases, thereby improving diagnostic when both tests are used together. Therefore, SCG may serve as a valuable complementary tool, and with additional validation and research, could in the future be used as an alternative to NT-proBNP.

### Optimizing patient selection for TTE Using SCG

TTE is a widely requested non-invasive cardiac imaging modality, and several studies have aimed to optimize its use to reduce waiting times and improve outcomes [10–14]. A risk classification based on the two SCG derived scores demonstrated SCG’s ability to identify patients at elevated risk of HF who may require further evaluation with TTE. Notably, SCG categorized 71 patients (51%) from the “No HF” group that was initially referred on the suspicion of HF, as minimal risk, potentially sparing them from unnecessary imaging. However, the remaining patients were classified as either at elevated risk of HF (41%) or elevated risk of low ejection fraction (8%), warranting further assessment.

Importantly, SCG successfully identified the need for TTE in almost all HF patients, misclassifying only six cases, five with HFpEF and one with HFrEF as minimal risk of HF. Although the algorithm missed five HFpEF cases, this diagnosis is known to be challenging to confirm, even with TTE.

These findings suggest that SCG may have potential as a complementary pre-TTE risk stratification tool. However, the clinical utility of such an approach requires an evaluation of the balance between reducing unnecessary TTE referrals and the risk of missed HF diagnoses. Prospective studies assessing clinical outcomes, resource utilization, and the benefits are therefore needed before SCG can be considered as a triage tool in routine practice.

### Strength and limitations

This study demonstrates the development of a robust SCG-based algorithm that performs very well in identifying HF. The SCG-based “AnyHF score” and “HFrEF-score” showed high diagnostic accuracy, with excellent sensitivity, negative predictive value, and strong AUC-ROC, indicating their potential as reliable noninvasive tools for HF detection. The algorithms were able to stratify risk even among patients referred for suspected HF but ultimately without HF, potentially reducing unnecessary further testing. Although the primary evaluation was based on a predefined sequential training/test split, the additional 5-fold cross-validation demonstrated consistent model performance, supporting the robustness of the developed algorithms. However, the study has some important limitations. First, both the training and test cohorts were relatively small. Future work should include external calibration and validation in independent cohorts to ensure reproducibility and generalizability. Second, patients with atrial fibrillation and implanted cardiac devices were excluded due to technical limitations, which limit applicability to the broader HF population. Third, the validation cohort consisted exclusively of patients with newly diagnosed HFrEF recruited to address the limited representation of HFrEF in the main study. As this cohort may represent a more selected clinical population than patients presenting with suspected HF, this could influence the reported performance estimates, particularly the PPV. Importantly, the validation cohort was used exclusively for independent testing of the locked algorithms and did not contribute to model development or threshold selection. Fourth, the results for HFmEF are limited, with only four patients included. Finally, patients with acute coronary syndrome as the primary cause of the current HF episode were excluded, though patients with prior ACS or chronic ischemic HF were eligible, maintaining a representative spectrum of HF etiologies.

## Conclusion

This study suggests that SCG has potential to aid in the diagnostic process of HF. The SCG-based algorithms “AnyHF-score” and “HFrEF-score” demonstrated high diagnostic performance. The HFrEF-score showed higher specificity and PPV than NT-proBNP. These findings indicate that SCG could serve as a complementary tool to potentially reduce false-positive cases and better identify patients at risk of HF. Future studies should validate these results in larger, more diverse, and externally confirmed cohorts.

## Supporting information

S1 Table Any HF-score and HFrEF-score in patient groups. (DOCX)

S2 Table Echocardiographic data. (DOCX)

S3 Table BNP Measurments. (DOCX)

S4 Table Performance of AnyHF-score to detect all types of heart failure. (DOCX)

S5 Table Performance of AnyHF-score to detect HFpEF. (DOCX)

S6 Table Performance of HFrEF-score to detect HFrEF versus No HF. (DOCX)

S7 Table Performance of NT-proBNP to detect HFrEF versus No HF. (DOCX)

S8 Table Performance of the SCG algorithms using 5-fold cross-validation in the development cohort. (DOCX)

S9 Table Enrollment periods in the predefined training and test cohorts by study site. (DOCX)

S10 Table Performance of HFrEF-score to detect all types of heart failure at study sites. (DOCX)

S11 Table Performance of AnyHF-score to detect all types of heart failure at study sites. (DOCX)

S1 Text Detailed Algorithm development and description. (DOCX)

S1 Fig Design of the clinical investigation. (TIFF)

S2 Fig Performance of the AnyHF-score to detect all HF vs no HF and HFpEF vs no HF. (TIFF)

S3 Fig Performance of the HFrEF-score to detect HFrEF vs no HF and HFrEF vs no HF. (TIFF)

S4 Fig Scatterplot of the two risk scores. (TIFF)
