# Supplementary material for: A feasibility study evaluating seismocardiography for the detection of heart failure
Source: PLOS Digit Health. 2026 Sep 10;5(9):e0001685. doi: 10.1371/journal.pdig.0001685 (PMC13561325; doi:10.1371/journal.pdig.0001685)
Supplement: Supplementary file 1 [file pdig.0001685.s001.docx]

| S1 Table. Any HF-score and HFrEF-score in patient groups | | | |  |  |
| --- | --- | --- | --- | --- | --- |
|  | **All** | | **No HF** | **HFrEF** | **HFpEF** |
| AnyHF-score, N | **208** | | **139** | **29** | **36** |
| Mean ± SD | 0.341±0.302 | | 0.219±0.221 | 0.683±0.278 | 0.497±0.283 |
| Min-Max | (0.00205-1) | | (0.00205-0.949) | (0.0326-1) | (0.018-0.98) |
| HFrEF score, N | **208** | **139** | | **29** | **36** |
| Mean ± SD | 0.0818±0.182 | | 0.0212±0.0616 | 0.384±0.292 | 0.0558±0.101 |
| Min-Max | (9.24e-05-0.977) | | (9.24e-05-0.594) | 0.356(0.144-0.674) | (0.000341-0.468) |

**S1 Table:** Presents the SCG-based scores “Any-HF score” and “HFrEF score” in all patient groups. Any-HF-score outputs a continuous value, dichotomized by a cut-off of 0.15. The HFrEF-score also produces a continuous value; however, it is dichotomized by a cut-off of 0.05
