# Supplementary material for: A feasibility study evaluating seismocardiography for the detection of heart failure
Source: PLOS Digit Health. 2026 Sep 10;5(9):e0001685. doi: 10.1371/journal.pdig.0001685 (PMC13561325; doi:10.1371/journal.pdig.0001685)
Supplement: Supplementary file 2 [file pdig.0001685.s002.docx]

| **S2 Table. Echocardiographic data** | | | |  |  |
| --- | --- | --- | --- | --- | --- |
|  | **All** | **No HF** | **HFrEF** | **HFmEF** | **HFpEF** |
| **LV diastolic internal diameter (mm), N** | **212** | **141** | **28** | **0** | **39** |
| **Mean ± SD** | 49.1 ± 8.2 | 47.3 ± 6.1 | 61.7 ± 8.6 | 51.2 ± 6.9 | 46.2 ± 6 |
| **Min-Max** | 33 – 83 | 35 – 65 | 46 – 83 | 44 – 59 | 33 – 58 |
| **Lvmass-BSA (g-m^2^), N** | **204** | **137** | **29** | **4** | **34** |
| **Mean ± SD** | 97.1 ± 40.7 | 83 ± 27.2 | 152.5 ± 49.6 | 142 ± 74.2 | 101.4 ± 28.1 |
| **Min-Max** | 30 – 312 | 30 – 165 | 71 – 312 | 81 – 240 | 32 – 167.1 |
| **LV diastolic volume – BSA (mL-m^2^), N** | **194** | **131** | **29** | **4** | **30** |
| **Mean ± SD** | 59.1 ± 22.7 | 52.8 ± 15 | 92.3 ± 31.1 | 66 ± 7 | 53.5 ± 13 |
| **Min-Max** | 27.3 – 151.2 | 27.3 – 94.8 | 45 – 151.2 | 56 – 72.2 | 32.1 – 90 |
| **LV systolic volume – BSA (mL-m^2^), N** | **194** | **131** | **29** | **4** | **30** |
| **Mean ± SD** | 30 ± 21.9 | 22.8 ± 11 | 68.2 ± 29.4 | 31.9 ± 12 | 24.4 ± 9.2 |
| **Min-Max** | 4.8 – 123.9 | 4.8 – 75.6 | 12.3 – 123.9 | 14.4 – 40.1 | 13.6 – 46.3 |
| **LA volumes – BSA (mL-m^2^), N** | **211** | **140** | **28** | **4** | **39** |
| **Mean ± SD** | 32.5 ± 12.3 | 27.9 ± 9.1 | 48.7 ± 12.5 | 35.6 ± 18 | 37.2 ± 10 |
| **Min-Max** | 13.6 – 81.4 | 13.7 – 81.4 | 28.6 – 78 | 17.5 – 60 | 13.6 – 66.1 |
| **LVEF (%), N** | **203** | **132** | **29** | **4** | **36** |
| **Mean ± SD** | 54 ± 13.3 | 58.8 ± 5.5 | 25.4 ± 9.6 | 47.8 ± 2.1 | 59.4 ± 5.9 |
| **Min-Max** | 12 – 77 | 40 – 77 | 12 – 56 | 46 – 50 | 50 – 71 |
| **MPI, N** | **205** | **142** | **22** | **4** | **37** |
| **Mean ± SD** | 0.5 ± 0.3 | 0.5 ± 0.2 | 1 ± 0.4 | 0.5 ± 0.2 | 0.4 ± 0.1 |
| **Min-Max** | 0.1 – 1.7 | 0.1 – 1.4 | 0.4 – 1.7 | 0.4 – 0.8 | 0.1 – 0.8 |
| **Global longitudinal strain (%), N** | **203** | **124** | **29** | **3** | **27** |
| **Mean ± SD** | -16.8 ± 5.4 | -18.5 ± 3.4 | -7.7 ± 4.2 | -13.9 ± 0.2 | -19.1 ± 4 |
| **Min-Max** | -27.8 – 0 | -27.8 - -9.9 | -21.6 – 0 | -14 - -13.7 | -25 - -9.9 |
| **Mitral decelerations time (msec), N** | **205** | **142** | **21** | **4** | **38** |
| **Mean ± SD** | 210.8 ± 57.9 | 214 ± 48.1 | 133.6 ± 49.8 | 208.8 ± 19.8 | 241.9 ± 62.2 |
| **Min-Max** | 62 – 464 | 92 – 381 | 62 – 231 | 186 – 231 | 167 – 464 |
| **Diastolic E-A ratio (%), N** | **206** | **145** | **19** | **4** | **38** |
| **Mean ± SD** | 1 ± 0.4 | 0.9 ± 0.3 | 1.4 ± 0.7 | 0.7 ± 0.3 | 0.9 ± 0.3 |
| **Min-Max** | 0.4 – 2.7 | 0.5 – 2.5 | 0.4 – 2.7 | 0.5 – 1.1 | 0.5 – 2.2 |
| **Septal E-e’, N** | **206** | **142** | **22** | **4** | **38** |
| **Mean ± SD** | 11 ± 5.2 | 9.5 ± 3 | 19.6 ± 8.1 | 15.7 ± 8 | 11.1 ± 3.9 |
| **Min-Max** | 3.7 – 37.5 | 3.7 – 24.9 | 9.7 – 37.5 | 7 – 26 | 5.3 – 18.4 |
| **RV TAPSE (mm), N** | **207** | **139** | **28** | **3** | **37** |
| **Mean ± SD** | 22.2 ± 4.1 | 22.9 ± 3.8 | 18.4 ± 4 | 19.7 ± 2.3 | 22.3 ± 3.8 |
| **Min-Max** | 10 – 35 | 14 – 35 | 10 – 29 | 17 – 21 | 12 – 29 |
| **Peak tricuspid regurgitant velocity (mmHg), N** | **111** | **70** | **17** | **2** | **22** |
| **Mean ± SD** | 21.5 ± 15 | 16.2 ± 13.6 | 33 ± 16.9 | 26 ± 14.1 | 29.2 ± 8.8 |
| **Min-Max** | 0 – 61 | 0 – 57 | 0 – 61 | 16 – 36 | 0 – 44 |

**S2 Table:** Echocardiography measurements
