# Supplementary material for: A feasibility study evaluating seismocardiography for the detection of heart failure
Source: PLOS Digit Health. 2026 Sep 10;5(9):e0001685. doi: 10.1371/journal.pdig.0001685 (PMC13561325; doi:10.1371/journal.pdig.0001685)
Supplement: Supplementary file 3 [file pdig.0001685.s003.docx]

| **S3 Table. BNP measurement** | | | |  |  |
| --- | --- | --- | --- | --- | --- |
|  | **All** | **No HF** | **HFrEF** | **HFmEF** | **HFpEF** |
| **Plasma NT-proBNP (pmol-L), N** | 203 | 143 | 17 | 4 | 39 |
| **Mean ± SD** | 50.3 ± 137.8 | 16.2 ± 32.9 | 344.6 ± 347.2 | 92 ± 57.3 | 43 ± 49.4 |
| **Min-Max** | 0.7 – 1,282.1 | 0.7 – 378.4 | 8.5 – 1,282.1 | 26.7 – 146 | 5.9 – 300.9 |

**S3 Table**: BNP measurements
