# Supplementary material for: A feasibility study evaluating seismocardiography for the detection of heart failure
Source: PLOS Digit Health. 2026 Sep 10;5(9):e0001685. doi: 10.1371/journal.pdig.0001685 (PMC13561325; doi:10.1371/journal.pdig.0001685)
Supplement: Supplementary file 4 [file pdig.0001685.s004.docx]

| S4 Table. Performance of AnyHF-score to detect all types of heart failure | | |  |
| --- | --- | --- | --- |
|  | **All** | **Train** | **Test** |
| No HF, N | 139 | 75 | 64 |
| HF*, N | 69 | 25 | 44 |
| Prevalence of HF, % | 33.2% | 25% | 40.7% |
| True negative | 74 | 46 | 28 |
| False negative | 7 | 3 | 4 |
| False positive | 65 | 29 | 36 |
| True positive | 62 | 22 | 40 |
| AUC, % (CI) | 83.3% (76.9-89.7) | 84.9% (74.8-94.9) | 82% (73.4-90.5) |
| Sensitivity, % (CI) | 89.9% (80.2-95.8%) | 88% (68.8-97.5%) | 90.9% (78.3-97.5%) |
| Specificity, % (CI) | 53.2% (44.6-61.7%) | 61.3% (49.4-72.4%) | 43.8% (31.4-56.7%) |
| Negative predictive value, % (CI) | 91.4% (83-96.5%) | 93.9% (83.1-98.7%) | 87.5% (71-96.5%) |
| Positive predictive value, % (CI) | 48.8% (39.9-57.8%) | 43.1% (29.3-57.8%) | 52.6% (40.8-64.2%) |
| Rule out, % (CI) | 38.9% (32.3-45.9%) | 49% (38.9-59.2%) | 29.6% (21.2-39.2%) |
| Rule in, % (CI) | 61.1% (54.1-67.7%) | 51% (40.8-61.1%) | 70.4% (60.8-78.8%) |
| Likelihood ratio positive | 1.922 | 2.276 | 1.616 |
| Likelihood ratio negative | 0.1906 | 0.1957 | 0.2078 |

**S4 Table:** *HF is defined as HFrEF, HFmEF and HFpEF combined. Negative predictive values, specificity, rule-out and likelihood ratio negative are calculated for ‘AnyHF-scores’ ≤0.15. Positive predictive values, sensitivity, rule-in and likelihood ratio positive are calculated for ‘Any HF-scores’ >0.15. CI: 95% confidence interval
