# Supplementary material for: A feasibility study evaluating seismocardiography for the detection of heart failure
Source: PLOS Digit Health. 2026 Sep 10;5(9):e0001685. doi: 10.1371/journal.pdig.0001685 (PMC13561325; doi:10.1371/journal.pdig.0001685)
Supplement: Supplementary file 5 [file pdig.0001685.s005.docx]

| S5 Table. Performance of AnyHF-score to detect HFpEF | | |  |
| --- | --- | --- | --- |
|  | **All** | **Train** | **Test** |
| No HF, N | 139 | 75 | 64 |
| HFpEF*, N | 36 | 19 | 17 |
| Prevalence of HFpEF, % | 20.6% | 20.2% | 21% |
| True negative | 74 | 46 | 28 |
| False negative | 5 | 3 | 2 |
| False positive | 65 | 29 | 36 |
| True positive | 31 | 16 | 15 |
| AUC, % (CI) | 78.4% (69-87.8) | 81.3% (68.9-93.6) | 78.2% (64.4-92) |
| Sensitivity, % (CI) | 86.1% (70.5-95.3%) | 84.2% (60.4-96.6%) | 88.2% (63.6-98.5%) |
| Specificity, % (CI) | 53.2% (44.6-61.7%) | 61.3% (49.4-72.4%) | 43.8% (31.4-56.7%) |
| Negative predictive value, % (CI) | 93.7% (85.8-97.9%) | 93.9% (83.1-98.7%) | 93.3% (77.9-99.2%) |
| Positive predictive value, % (CI) | 32.3% (23.1-42.6%) | 35.6% (21.9-51.2%) | 29.4% (17.5-43.8%) |
| Rule out, % (CI) | 45.1% (37.6-52.8%) | 52.1% (41.6-62.5%) | 37% (26.6-48.5%) |
| Rule in, % (CI) | 54.9% (47.2-62.4%) | 47.9% (37.5-58.4%) | 63% (51.5-73.4%) |
| L | 1.841 | 2.178 | 1.569 |
| Likelihood ratio negative | 0.2609 | 0.2574 | 0.2689 |

**S5 Table:** Negative predictive values, specificity, rule-out and likelihood ratio negative are calculated for the SCG-based ‘AnyHF-scores’ ≤0.15. Positive predictive values, sensitivity, rule-in and likelihood ratio positive are calculated for ‘AnyHF-scores’ >0.15.

CI: 95% confidence interval
