# Supplementary material for: A feasibility study evaluating seismocardiography for the detection of heart failure
Source: PLOS Digit Health. 2026 Sep 10;5(9):e0001685. doi: 10.1371/journal.pdig.0001685 (PMC13561325; doi:10.1371/journal.pdig.0001685)
Supplement: Supplementary file 6 [file pdig.0001685.s006.docx]

| S6 Table. Performance of HFrEF-score to detect HFrEF versus No HF | | | |
| --- | --- | --- | --- |
|  | **All** | **Train** | **Test** |
| No HF, N | 139 | 75 | 64 |
| HFrEF, N | 29 | 4 | 25 |
| Prevalence of HFrEF, % | 17.3% | 5.06% | 28.1% |
| True negative | 128 | 71 | 57 |
| False negative | 3 | 1 | 2 |
| False positive | 11 | 4 | 7 |
| True positive | 26 | 3 | 23 |
| AUC, % (CI) | 93.4% (87-99.8) | 94.3% (78.4-100%) | 93.6% (86.8-100%) |
| Sensitivity, % (CI) | 89.7% (72.6-97.8%) | 75% (19.4-99.4%) | 92% (74-99%) |
| Specificity, % (CI) | 92.1% (86.3-96%) | 94.7% (86.9-98.5%) | 89.1% (78.8-95.5%) |
| Negative predictive value, % (CI) | 97.7% (93.5-99.5%) | 98.6% (92.5-100%) | 96.6% (88.3-99.6%) |
| Positive predictive value, % (CI) | 70.3% (53-84.1%) | 42.9% (9.9-81.6%) | 76.7% (57.7-90.1%) |
| Rule out, % (CI) | 78% (70.9-84%) | 91.1% (82.6-96.4%) | 66.3% (55.5-76%) |
| Rule in, % (CI) | 22% (16-29.1%) | 8.86% (3.64-17.4%) | 33.7% (24-44.5%) |
| Likelihood ratio positive | 11.33 | 14.06 | 8.411 |
| Likelihood ratio negative | 0.1123 | 0.2641 | 0.08982 |

**S6 Table:** Negative predictive values, specificity, rule-out and likelihood ratio negative are calculated for the SCG-based ‘HFrEF-score’ ≤0.05. Positive predictive values, sensitivity, rule-in and likelihood ratio positive are calculated for ‘HFrEF-score’ >0.05. CI: 95% confidence interval
