# Supplementary material for: A feasibility study evaluating seismocardiography for the detection of heart failure
Source: PLOS Digit Health. 2026 Sep 10;5(9):e0001685. doi: 10.1371/journal.pdig.0001685 (PMC13561325; doi:10.1371/journal.pdig.0001685)
Supplement: Supplementary file 7 [file pdig.0001685.s007.docx]

| **S7 Table. Performance of NT-proBNP to detect HFrEF versus No HF** | | | |
| --- | --- | --- | --- |
|  | **All** | **Train** | **Test** |
| **No HF, N** | 140 | 74 | 66 |
| **HFrEF, N** | 17 | 4 | 13 |
| **Prevalence of HFrEF, %** | 10.80% | 5.13% | 16.5% |
| **True negative** | 96 | 53 | 43 |
| **False negative** | 1 | 1 | 0 |
| **False positive** | 44 | 21 | 23 |
| **True positive** | 16 | 3 | 13 |
| **AUC, % (CI)** | 94.3% (86.6-100%) | 80.6% (54.3-100%) | 99.2% (95.7-100%) |
| **Sensitivity, % (CI)** | 94.1% (71.3-99.9%) | 75% (19.4-99.4%) | 100% (75.3-100%) |
| **Specificity, % (CI)** | 68.6% (60.2-76.1%) | 71.6% (59.9-81.5%) | 65.2% (52.4-76.5%) |
| **Negative predictive value, % (CI)** | 99% (94.4-100%) | 98.1% (90.1-100%) | 100% (91.8-100%) |
| **Positive predictive value, % (CI)** | 26.7% (16.1-39.7%) | 12.5% (2.66-32.4%) | 36.1% (20.8-53.8%) |
| **Rule out, % (CI)** | 61.8% (53.7-69.4%) | 69.2% (57.8-79.2%) | 54.4% (42.8-65.7%) |
| **Rule in, % (CI)** | 38.2% (30.6-46.3%) | 30.8% (20.8-42.2%) | 45.6% (34.3-57.2%) |
| **Likelihood ratio positive** | 2.995 | 2.643 | 2.87 |
| **Likelihood ratio negative** | 0.08578 | 0.3491 | 0 |

**S7 Table**: Negative predictive values, specificity, rule-out and likelihood ratio negative are calculated for NTpro-BNP ≤ 125pg-ml. Positive predictive values, sensitivity, rule-in and likelihood ratio positive are calculated for pro-BNP > 125pg-ml. CI: 95% confidence interval
