# Supplementary material for: A feasibility study evaluating seismocardiography for the detection of heart failure
Source: PLOS Digit Health. 2026 Sep 10;5(9):e0001685. doi: 10.1371/journal.pdig.0001685 (PMC13561325; doi:10.1371/journal.pdig.0001685)
Supplement: Supplementary file 8 [file pdig.0001685.s008.docx]

**S8 Table. Performance of the SCG algorithms using 5-fold cross-validation in the development cohort**

|  | Macro AUC | Micro AUC | Weighted AUC |
| --- | --- | --- | --- |
| HFrEF-score vs all other patients | 0.850 | 0.965 | 0.877 |
| HFrEF-score vs No HF | 0.900 | 0.967 | 0.912 |
| AnyHF-score vs No HF | 0.833 | 0.878 | 0.835 |
