# Supplementary material for: A feasibility study evaluating seismocardiography for the detection of heart failure
Source: PLOS Digit Health. 2026 Sep 10;5(9):e0001685. doi: 10.1371/journal.pdig.0001685 (PMC13561325; doi:10.1371/journal.pdig.0001685)
Supplement: Supplementary file 9 [file pdig.0001685.s009.docx]

**S9 Table. Enrollment periods in the predefined training and test cohorts by study site**

|  | Aalborg site | Odense site | Validation cohort |
| --- | --- | --- | --- |
| Training set enrollment period | 29-jun.2018 to  4-apr.2019 | 3-seb.2018 to  12-aug.2019 |  |
| Test set enrollment period | 26-apr.2019 to  3-april.2020 | 26-aug.2019 to  20-feb.2020 | 27-jun.2021 to  27-jul.2022 |

**S9 Table:** Enrollment periods for the predefined training and test cohorts at the Aalborg and Odense study sites, and for the independent HFrEF validation cohort.
