# Supplementary material for: A feasibility study evaluating seismocardiography for the detection of heart failure
Source: PLOS Digit Health. 2026 Sep 10;5(9):e0001685. doi: 10.1371/journal.pdig.0001685 (PMC13561325; doi:10.1371/journal.pdig.0001685)
Supplement: Supplementary file 10 [file pdig.0001685.s010.docx]

| **S10 Table. Performance of HFrEF-score to detect all types of heart failure at study sites.** | | |
| --- | --- | --- |
| HFrEF-score | **Aalborg** | **Odense** |
| No HFrEF (n) | 82 | 57 |
| HFrEF (n) | 27 | 2 |
| Prevalence of true | 24.8% | 3.39% |
| AUC | 93.5% (86.9-100) | 99.1% (89.9-108) |
| Negative predictive value (HFrEF-score<=0.05 | 96.2% (89.3-99.2%) | 100% (93.2-100%) |
| Positive predictive value (HFrEF-score>0.05) | 80% (61.4-92.3%) | 28.6% (3.67-71%) |
| Sensitivity (HFrEF-score>0.05) | 88.9% (70.8-97.6%) | 100% (15.8-100%) |
| Specificity (HFrEF-score<=0.05) | 92.7% (84.8-97.3%) | 91.2% (80.7-97.1%) |
| Rule out (HFrEF-score<=0.05) | 72.5% (63.1-80.6%) | 88.1% (77.1-95.1%) |
| Rule in (HFrEF-score>0.05) | 27.5% (19.4-36.9%) | 11.9% (4.91-22.9%) |
| Likelihood ratio positive (HFrEF-score>0.05) | 12.15 | 11.4 |
| Likelihood ratio negative (HFrEF-score<=0.05) | 0.1199 | 0 |

**S10 Table:** Negative predictive values, specificity, rule-out and likelihood ratio negative are calculated for the SCG-based ‘HFrEF-score’ ≤0.05. Positive predictive values, sensitivity, rule-in and likelihood ratio positive are calculated for ‘HFrEF-score’ >0.05.
