# Supplementary material for: A feasibility study evaluating seismocardiography for the detection of heart failure
Source: PLOS Digit Health. 2026 Sep 10;5(9):e0001685. doi: 10.1371/journal.pdig.0001685 (PMC13561325; doi:10.1371/journal.pdig.0001685)
Supplement: Supplementary file 11 [file pdig.0001685.s011.docx]

**S11 Table. Performance of AnyHF-score to detect all types of heart failure at study sites**

| AnyHF-score | **Aalborg** | **Odense** |
| --- | --- | --- |
| No HF (n) | 82 | 57 |
| HF (n) | 35 | 15 |
| Prevalence of True | 29.9% | 20.8% |
| AUC | 84.6% (76-93.2) | 77.3% (62.5-92.2) |
| Negative predictive value (AnyHF-score <=0.15) | 91.7% (80-97.7%) | 96.8% (83.3-99.9%) |
| Positive predictive value (AnyHF-score >0.15) | 44.9% (32.9-57.4%) | 34.1% (20.1-50.6%) |
| Sensitivity (AnyHF-score >0.15) | 88.6% (73.3-96.8%) | 93.3% (68.1-99.8%) |
| Specificity (AnyHF-score <=0.15) | 53.7% (42.3-64.7%) | 52.6% (39-66%) |
| Rule out (AnyHF-score <=0.15) | 41% (32-50.5%) | 43.1% (31.4-55.3%) |
| Rule in (AnyHF-score >0.15) | 59% (49.5-68%) | 56.9% (44.7-68.6%) |
| Likelihood ratio positive (AnyHF-score >0.15) | 1.911 | 1.97 |
| Likelihood ratio negative (AnyHF-score <=0.15) | 0.213 | 0.1267 |

**S11 Table:** Negative predictive values, specificity, rule-out and likelihood ratio negative are calculated for the SCG-based ‘AnyHF-score’ ≤0.15. Positive predictive values, sensitivity, rule-in and likelihood ratio positive are calculated for ‘HFrEF-score’ >0.15.
