# Supplementary material for: A feasibility study evaluating seismocardiography for the detection of heart failure
Source: PLOS Digit Health. 2026 Sep 10;5(9):e0001685. doi: 10.1371/journal.pdig.0001685 (PMC13561325; doi:10.1371/journal.pdig.0001685)
Supplement: Supplementary file 12 [file pdig.0001685.s012.docx]

**S1 Text:**

**Detailed Algorithm development and description**

**Pre-processing**

The current methods rely on modified pre-processing steps from the CAD-score system, which was originally developed for Heart sound recordings.

1. Segmentation of the SCG signal into systolic and diastolic periods
2. Rejection of noise heartbeats
3. Construction of an ensemble average signal
4. Identify the systolic fiducial points G_s_ and the diastolic fiducial point D_d_ in the ensemble average SCG, using fiducial point detection algorithm.

**Autoencoder algorithm**

Two autoencoder algorithms are developed, one for the systolic complex and one for the diastolic complex.

1. Selection of an analyzing segment around the G_s_ fiducial point in the systolic complex and segment around the D_d_ fiducial point in the diastolic complex.
2. Pass the SCG segments into the pre-trained autoencoders and predict the same SCG segments using the autoencoders. Note the autoencoders were trained in only SCG’s from healthy subjects.
3. Compare the similarity between the predicted SCG with the actual SCG segment, using Pearson correlation coefficient.
   a. rSys: is the correlation between the actual SCG systolic segment and the predicted SCG systolic segment.
   b. rDia: is the correlation between the actual SCG diastolic segment and the predicted SCG diastolic segment.

The main principle of the above method is the SCG signal is compressed down to very few variables (Nodes) in the autoencoder, from the compressed version the autoencoder the reconstructs (predicts) the SCG signal. Since the autoencoder is trained in healthy subjects, it’s efficient in reconstruction of normal SCG’s, but less efficient in the reconstruction of more complex SCG signals such as HF signals.

Both autoencoders are single layers, and the number of nodes was 8 for both autoencoders.

**Training and validation of autoencoders**

The dataset was divided into training and test-set. The autoencoders were only trained in the non-HF patients on the training-set. Since the autoencoders were not trained to detect HFrEF, the autoencoders can be considered as an unsupervised machine learning method.

**Deviations from the clinical investigation plan**

| **Deviation** | **Description of deviation** |
| --- | --- |
| Data not split in development and validation sets as per protocol | The additional 20 HFrEF patients from the extension study were not split 50-50 into development and validation sets as originally described in the protocol. Instead, all 20 were included in the validation set. This decision was based on the observation that the algorithm, developed on the main study population, already presented excellent performance with a high AUC, and was therefore not expected to benefit from additional training. From a statistical point of view, this could only bias the results in the direction of less superior performance in the validation set, as the algorithm was not trained to detect the HFrEF patients from the extension study.  Among patients having both a valid xiphoid SCG recording and a HF diagnosis, the first 100 patients enrolled in the main study were used in the development set, and the last 89 patients enrolled in the main study were used in the validation set. |
| The per protocol definition of ‘healthy subjects’ is not followed: healthy subjects’=Not having any of the definitions of HF (HFrEF, HFmEF and HFpEF) or having heart valve disease detected by TTE. | The definition of healthy subjects was not used. Instead, the definition ‘no HF’ was used, defined as subjects not having any of the definitions of HF (HFrEF, HFmEF and HFpEF).  This was because the term “healthy” is unprecise wording since the patients without HF might suffer from other morbidities. |
| Patient included in algorithm development as No HF patient despite uncertainty on final HF diagnosis | For one patient the final HF diagnosis was uncertain due to missing pro-BNP data but was included in the training set for algorithm development as a ‘No HF’ patient by mistake.  The patient had a LVEF=55% and left atrial volume index = 35 and thus, dependent on proBNP value, could have been classified as HFpEF patient. The patient could not have been classified as HFrEF and HFmEF, as the echocardiography data did not support these diagnoses.  The potential consequence is that the development of the ‘AnyHF-score’ could have been impacted. The patient was included in the training set as No HF in the development of the ‘AnyHF-score’. If the patient instead had a true HF diagnosis as HFpEF, this deviation would have impacted the development of the score.  The ’HFrEF-score’ is not affected by this deviation. |
| One patient with No-HF misclassified | One patient was initially classified as having HFrEF; however, the individual presented with NYHA class I symptoms, an ejection fraction of 56%, and normal TTE parameters, thus indicating no heart failure. Including this patient in the HFrEF category would only impair the performance of the SCG algorithms rather than improve them. |
